# The Privilege of Advancing Knowledge

**DOI:** 10.31486/toj.25.5055

**Published:** 2025

**Authors:** Ronald G. Amedee

**Affiliations:** Director of Clinical School and Professor, The University of Queensland Medical School, Ochsner Clinical School, New Orleans, LA; Editor-in-Chief, *Ochsner Journal*

*The advancement and diffusion of knowledge … is the only guardian of true liberty.*

–James Madison, 4^th^ US President

The Summer 2025 issue of the *Ochsner Journal* contains four original research articles, one letter to the editor, an innovative program submission, and eight case reports and clinical observations. The letter to the editor is entitled “Racial Differences in Radium-223 Treatment Response and Adverse Effects in a Small-Cohort, Pilot, Hypothesis-Generating Observational Study” and is authored by Yang, Nittala, S Vijayakumar, and V Vijayakumar. Srinivasan and Vani Vijayakumar are Ochsner Health faculty members in the Department of Radiology. The innovative program submission is entitled “Developing an Ownership Model for Experiential Learning of Social Determinants of Health for Medical Residents” and is by Ochsner Health authors Noor, Rahat, George, Capuchina, Ruiz, Broussard, and Laborde. The senior author on the manuscript, Dr Yvens Laborde, currently serves as Chief Community Medical Officer for Ochsner Health. Yvens frequently submits articles for publication in this journal, and as a key leader in the Ochsner Health Division of Academics, he frequently educates and mentors learners throughout the medical academic continuum of undergraduate medical education and graduate medical education.

Our first original research article is written by the team of Mosley, Davis, and Truax who review “HEART Score Agreement Between Attending and Resident Emergency Medicine Physicians for Patients With Potential Acute Coronary Syndrome.” The second original research article details the “Accuracy of Perfusion Index and Perfusion Index Ratio as a Predictor of a Successful Low Interscalene Brachial Plexus Block: A Prospective Observational Study” and is authored by Jain, Srinivas, Ahmad, Kaushal, Kumar, and Waindeskar. “3D-Printed Patient-Specific Models of the Aortic Arch for Advanced Visualization of Complex Neurointerventional Cases” is submitted by authors Mahapatra, Bhimarasetty, Rahim, Curtis, Gulotta, and Sarkar. Several of the authors of this paper are affiliated with Ochsner Health: Dr Gulotta is an interventional radiologist, Dr Sarkar is the director of the Ochsner Health BioDesign Lab, Mr Curtis is a biomedical engineer in the BioDesign Lab, and Mr Rahim is a University of Queensland-Ochsner Clinical School student. Completing this section is an article by Mauras, McMahon, Petrie, Roubion, Bronstone, Leonardi, and Dasa on “Reduction in Opioid Requirements Following Changes to Regional Anesthesia for Patients Undergoing Total Knee Arthroplasty.”

The case reports and clinical observations section includes “Rectal Administration of Carbidopa/Levodopa” by Ochsner Health authors Guthrie, King, and Casey, followed by another Ochsner Health–authored report entitled “HeartMate 3 Left Ventricular Assist Device Implantation in a Pediatric Patient With Limb-Girdle Muscular Dystrophy” by Zumwalt, Boucek, and Wells. The next report, entitled “Jarcho-Levin Syndrome With Fatal Respiratory Failure,” is authored by Atitallah, Othmen, Rabeh, Missaoui, Bouyahia, Mazigh, Yahyaoui, and Boukthir. The next case is a report on “Late Pancreatic Metastasis From Papillary Thyroid Carcinoma Diagnosed by Endoscopic Ultrasound-Guided Tissue Acquisition” written by Lopes, Pereira-Lima, de Souza Salerno, and Luzzatto, followed by “Rare Ocular Association With Hailey-Hailey Disease” written by Singh, Pathania, and Mishra, and “Use of Endoscopic Scissors to Remove a Foreign Body Impacted in the Proximal Esophagus” submitted by Mubarak. Our final two reports are “Incidental Finding of an Infarcted Epiploic Appendage Attached to the Sigmoid Colon” by Uche and Hirsch and “Bilateral Hypoglossal Nerve Palsy After Cardiac Surgery” authored by the Ochsner Health team of Vining, Trusheim, and Ural.

This edition of *Ochsner Journal* will be published a few weeks prior to the 2025 July 4^th^ holiday weekend marking the 249^th^ commemoration of the adoption of the Declaration of Independence in 1776 that declared our independence from British rule. The day is typically celebrated with pomp and parades; backyard barbeques; patriotic gatherings of family, close friends, and community neighbors; and finally, a sparkling evening of fireworks. A quick search reveals numerous powerful quotes that inspire our appreciation for and reflection on the freedoms we as Americans enjoy each day and that also honor those who have fought to protect our freedoms. The quote above from James Madison reminds all who serve in health care that our ability to advance and spread knowledge to our patients, learners, and one another is a distinct privilege, liberty, and freedom that we enjoy. The freedom to publish and disseminate this and many other medical journals is yet another such example of true liberty. Happy July 4^th^ to each of our readers and let us begin to plan celebrations on an even more grand scale to mark 250 years of independence in 2026!

